# Preparation and characterization of immobilized indole−3−butyric acid on silver−cerium oxide nanoparticles for improving its stability and utilization in the study of plant root formation indices

**DOI:** 10.3389/fpls.2025.1570945

**Published:** 2025-06-06

**Authors:** Masoumeh Ghorbani, Danial Kahrizi, Elham Arkan, Zhennan Yu

**Affiliations:** ^1^ Department of Nanobiotechnology, Faculty of Strategic Sciences and Technologies, Razi University, Kermanshah, Iran; ^2^ Department of Biotechnology, Faculty of Agriculture, Tarbiat Modares University, Tehran, Iran; ^3^ Nano Drug Delivery Research Center, Health Technology Institute, Kermanshah University of Medical Sciences, Kermanshah, Iran; ^4^ Department of Anatomy, Jeonbuk National University Medical School, Jeonju, Republic of Korea

**Keywords:** Ag@CeO2 nanoparticles, agricultural nanotechnology, nanocarrier, Nicotiana tabacum, rooting, tissue culture

## Abstract

**Introduction:**

In recent years, nanocarriers have emerged as sophisticated platforms for the encapsulation and targeted delivery of agricultural chemicals, with particular emphasis on auxins. Owing to their remarkable potential to enhance bioavailability and optimize crop productivity, these nanotechnological systems have garnered considerable scientific interest.

**Methods:**

Here, the synthesis and efficacy of silver-cerium oxide (ceria) nanoparticles (Ag@CeO2 NPs) as a metal-oxide nanocarrier for loading the hormone indole-3-butyric acid (IBA) and its effect on tobacco plant rooting were investigated. Tobacco plant stems were cultured in different concentrations of IBA−Ag@CeO2 NPs (1, 2, and 3 mgL-1) in plant culture medium, and rooting indices were evaluated after 4 weeks.

**Results and Discussion:**

The mean particle size (MPS) of IBAloaded Ag@CeO₂ nanoparticles was determined to be 389 nm via dynamic light scattering (DLS) analysis. Scanning electron microscopy (SEM) further revealed that the nanoparticles possessed a predominantly spherical morphology, with a mean diameter of approximately 136 nm. The successful immobilization of IBA onto the Ag@CeO₂ nanocarriers was confirmed by Fourier-transform infrared spectroscopy (FTIR), which identified the characteristic absorption peaks of the hormone on the nanoparticle surface. Moreover, the IBA loading process achieved a hormone loading efficiency (HLE) of 45% and an encapsulation efficiency (EE) of approximately 90%. The results showed that IBA−Ag@CeO2 NPs at a concentration of 2 mgL-1 had the highest efficiency in promoting the rooting of tobacco (*Nicotiana tabacum*) plants compared with other treatments. Our results suggest that Ag@CeO2 NPs function as an effective nanocarrier for delivering the IBA hormone, highlighting their potential applications in agricultural practices.

## 1 Introduction

Nanobiotechnology has rapidly emerged as a multidisciplinary frontier, integrating the principles of nanoscience and biotechnology to unlock novel, biology-driven applications. Nanomaterials, characterized by their unique one-dimensional or multi-dimensional architectures, have found widespread utility across a spectrum of scientific and technological domains. The synergistic convergence of nanotechnology and biotechnology has catalyzed transformative advancements in human well-being, with profound implications for sectors such as agriculture. Nanomaterials are typically classified as natural, incidental, or engineered, with the latter category-engineered nanomaterials-garnering considerable attention due to their tailored physicochemical properties and versatile applications in medicine, materials science, and agriculture. In the agricultural context, engineered nanoparticles have been strategically employed as “nanocarriers,” enabling the precise delivery of herbicides, agrochemicals, or genetic material to targeted plant tissues. This targeted delivery enhances the efficacy of active agents, facilitates controlled release, and ultimately contributes to the optimization and sustainability of modern agricultural practices (Ghorbani and Kahrizi, 2024; Lian et al., 2012; Zakaria et al., 2015). Nanotechnology is one of the advanced technologies in agricultural science because it enables material manipulation at the microscale compared to the micro- or macro scale (Kandasamy and Maity, 2015; Sukumaran et al., 2018). A diverse array of nanoparticles has been synthesized and systematically evaluated for their multifaceted effects on plant tissue culture systems. These investigations have encompassed the mitigation of fungal and bacterial contamination (Abdi et al., 2008; Safavi, 2014), the enhancement of shoot proliferation (Aghdaei et al., 2012), the promotion of somatic embryogenesis and subsequent regeneration (Chutipaijit, 2015), the induction of callus formation (Amiri et al., 2016), the stimulation of root development (Wang et al., 2016; Thangavelu et al., 2018), and the modulation of leaf physiological activity (Pereira et al., 2017). Previous studies have demonstrated that the encapsulation of agrochemicals, such as herbicides, within nanoparticulate carriers significantly enhances the efficacy of weed control while simultaneously improving environmental safety (Oliveira et al., 2015). Moreover, it has been proposed that biodegradable nanoformulations not only facilitate superior permeability through plant micro-cuttings but also enable the controlled and targeted release of active agents upon reaching the intended plant tissues (Santo Pereira et al., 2017; Karakeçili et al., 2019). In addition to their delivery capabilities, nanoparticles inherently possess antimicrobial, antifungal, and insecticidal properties, thereby contributing to effective disease management and ultimately enhancing agricultural productivity (Woodward and Bartel, 2005; Nuzzo et al., 2014).

Cerium (Ce), a lanthanide element distinguished by its 4f electron configuration, has garnered significant attention from researchers across the disciplines of physics, chemistry, biology, and materials science. In particular, cerium oxide (CeO₂), which adopts a stable fluorite crystal structure in nanoparticle formulations, exhibits unique physicochemical characteristics due to its oxygen-rich lattice (Conesa, 1995). Notably, CeO₂ nanoparticles possess intrinsic oxidase-mimetic activity, rendering them exceptionally valuable for a wide array of biological applications, including bioanalysis, agriculture, biomedicine, drug delivery, and tissue engineering scaffolds. These multifunctional properties position cerium oxide as a material of considerable scientific intrigue and commercial promise (Karakoti et al., 2010; Mandoli et al., 2010; Celardo et al., 2011; Li et al., 2013; Xu et al., 2013). Ag@CeO₂ NPs represent a highly promising platform for advancing agricultural productivity and sustainability. These nanostructures enable the targeted delivery of essential nutrients, thereby significantly enhancing their bioavailability and uptake by plants. Furthermore, the application of Ag@CeO₂ NPs as nanopesticides offers the potential to reduce dependence on conventional chemical pesticides, thus fostering more environmentally sustainable agricultural practices (Upadhyaya et al., 2023). At subtoxic concentrations, cerium oxide nanoparticles (CeO₂ NPs) have been shown to stimulate plant growth and physiological processes, as well as to activate stress-responsive signaling pathways that facilitate the scavenging of reactive oxygen species (ROS) generated under adverse conditions (Viswanathan et al., 2024). In addition, silver nanoparticles (Ag NPs) are well-documented for their potent antibacterial, antifungal, and antiviral activities, which contribute to effective disease management and improved crop yields, ultimately enhancing overall plant resilience. Despite these substantial benefits, it is imperative to address potential concerns regarding the toxicity of Ag@CeO₂ NPs to non-target organisms and the broader environment. Comprehensive risk–benefit assessments are therefore essential to ensure the safe and responsible deployment of these nanomaterials in agricultural systems (Kumari and Singh, 2016).

**Figure 1:**
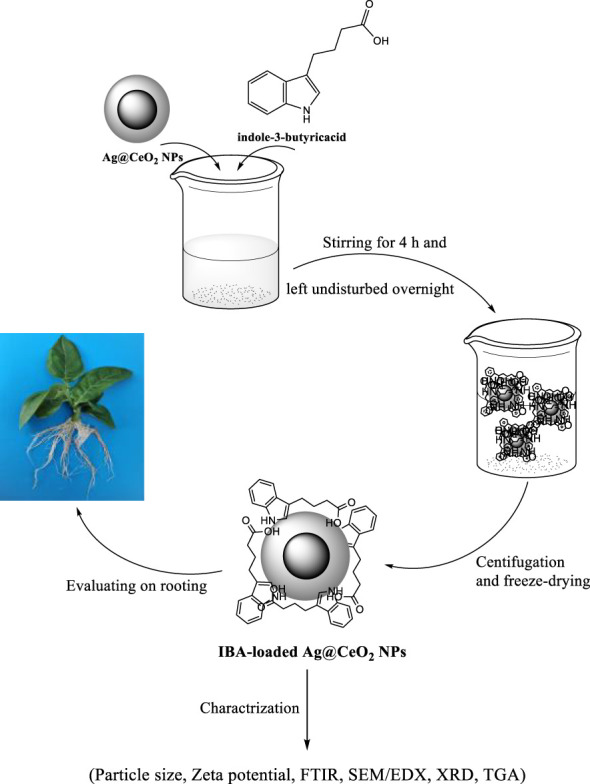
Schematic illustration for preparation of hormone indole−3−butyric acid loaded on silver−ceria nanoparticles (IBA−Ag@CeO_2_ NPs) and biological application.

Auxins are a class of phytohormones that play a pivotal role in regulating plant growth and orchestrating the formation of adventitious roots (Nuzzo et al., 2014; Labeeuw et al., 2016). In horticultural practice, insufficient root development remains a persistent challenge, with substantial economic repercussions-accounting for the annual loss of 10–25% of nursery plants and approximately 5% of ornamental species worldwide. Although exogenous application of auxins has been widely adopted to stimulate rooting and facilitate the propagation of cuttings, this approach frequently yields inconsistent outcomes, particularly in recalcitrant species or genotypes. Recent advances in nanotechnology have introduced alternative strategies for rooting, offering promising solutions to these limitations. Nanotechnology-enabled rooting techniques not only enhance the rooting efficiency of otherwise difficult-to-propagate species but also present a cost-effective, straightforward, and safe management strategy for improving overall plant propagation success (Thangavelu et al., 2018). IBA is a pivotal plant growth regulator that plays an essential role in stimulating root development and facilitating seed germination across a diverse range of plant species. Although IBA exhibits greater chemical stability than indole-3-acetic acid (IAA), both auxins are inherently susceptible to degradation over time. Notably, IBA is transported within plant tissues at a slower rate compared to IAA, a characteristic that may constrain its efficacy in promoting root formation (Epstein and Ludwig-Müller, 1993). Furthermore, the rapid metabolic conversion of IBA in planta-primarily through conjugation-can significantly diminish its sustained bioactivity, thereby reducing its availability for effective root induction when required (Damodaran and Strader, 2019). These limitations underscore the pressing need for environmentally benign formulations that can mitigate auxin degradation and minimize losses, as emphasized in previous research (Dong et al., 2018). Despite advances in plant tissue culture (PTC) techniques, recalcitrant genotypes have yet to consistently benefit from conventional auxin-based rooting protocols. Recent investigations have demonstrated that nanoscale formulations offer a promising alternative for enhancing rooting efficiency. For example, Shlar and Poverenov (2021) reported that encapsulation of IAA within magnesium–aluminum layered double hydroxide matrices markedly improved adventitious root formation and development in mung bean (*Vigna radiata* (L.) *Wilczek*), attributable to the protection of IAA from enzymatic degradation and its sustained, controlled release from the nanocarrier. Such findings highlight the transformative potential of nanotechnology-enabled delivery systems in overcoming the intrinsic limitations of traditional auxin applications. Clemente et al. (2019) introduced an environmentally benign, lipid-based nanocarrier system for the co-delivery of IBA and naphthaleneacetic acid (NAA), utilizing olive pomace in combination with two distinct phospholipids to formulate the nanoencapsulated hormones. The efficacy of this innovative nanocarrier was evaluated using olive tree cuttings from two cultivars: ‘*Leccino*,’ characterized by facile rooting, and ‘*Leccio del Corno*,’ known for its recalcitrance to root induction. Their findings revealed that application of the nanocarrier, in conjunction with both auxins, resulted in a pronounced increase in both root number and length across both cultivars. Given the pivotal role of the rooting stage in micropropagation-and the substantial economic losses that may arise from unsuccessful root induction-there is a critical need for novel strategies to enhance rooting efficiency. The integration of nanotechnology with micropropagation has thus emerged as a highly promising approach to overcome these limitations (Kashyap et al., 2015). In light of these advances, the present study was designed to prepare indole-3-butyric acid immobilized on Ag@CeO₂ NPs and to systematically evaluate its efficacy in promoting root formation in tobacco (*Nicotiana tabacum*) plants (**Figure 1**).

## 2 Materials and methods

The chemicals were procured from Fluka (Buchs, Switzerland), Sigma−Aldrich (St. Louis, MO, USA) and Merck (Darmstadt, Germany) corporations and used as received without undergoing additional purification procedures. For the average size, the hydrodynamic diameter was measured using a dynamic light scattering (DLS) (Malvern Zetasizer Nano S90) instrument. The surface charge of IBA−loaded Ag@CeO_2_ NPs was evaluated using Zeta potential measurement. Morphology and particle size distribution were analyzed from multiple fields of view. Freeze-dried nanoparticles were mounted on aluminum stubs using double-sided carbon tape and sputter-coated with a thin layer of gold to enhance conductivity. Scanning electron microscopy (SEM) images were obtained using an SEM equipped with an energy-dispersive X−ray analyzer (EDAX) (Quanta 450, FEI,USA) at an accelerating voltage of 20 kV. Powdered samples were analyzed using a Bruker D8 Advance X-ray diffractometer with Cu Kα radiation (λ = 1.54050 Å) operated at 40 kV and 40 mA. Data were collected over a 2θ range of 10° to 80° with a step size of 0.02° and scan speed of 1°/min. Phase identification was performed by comparing diffraction peaks with standard JCPDS reference patterns. Samples (IBA, Ag@CeO₂ NPs, and IBA-loaded Ag@CeO₂ NPs) were ground with KBr and pressed into pellets. FTIR spectra were recorded on a Shimadzu Prestige-21 spectrometer in the range of 400–4000 cm^−1^ with a resolution of 4 cm^−1^ and 32 scans per sample. A Setaram instrument was used to perform thermogravimetric analysis (TGA) (STA6000, PerkinElmer, Waltham, MA, USA) in a nitrogen atmosphere, with a 10°C/min heating rate over a 30-650°C temperature range. Encapsulation efficiency (EE) measures the percentage of nanoparticles that successfully encapsulate drugs. On the other hand, hormone loading efficiency (HLE) is the amount of drug loaded per unit weight of the nanoparticle.%EE and %HLE were measured using Equations 1 and 2 (Korpayev et al., 2021).


(1) $$HLE\;=\;\left[total\;amount\;of\;hormone\;-\;amount\;of\;hormone\;in\;supernatant/total\;amount\;of\;nanoparticle\;recovered\right]\;\times \;100$$

(2) $$EE\;=\;\left[total\;amount\;of\;hormone\;-\;amount\;of\;hormone\;in\;supernatant/total\;amount\;of\;hormone\right]\;\times \;100$$

The controlled release of IBA from Ag@CeO_2_ NPs was studied in the phosphate-buffered saline (PBS) medium using a dialysis bag method with a cellulose membrane tube (molecular weight cutoff: 12 kDa). All experiments were conducted in triplicate, and the findings were subsequently reported. The experimental data were subjected to analysis utilizing SPSS software. We have conducted a comprehensive statistical analysis using the analysis of variance (ANOVA) and *post-hoc* Tukey’s Honest Significant Difference (HSD) tests to compare the mean root length, dry and fresh root weights, and day to rooting among the different concentrations of IBA−Ag@CeO_2_ NPs.

### 2.1 Preparation of Ag@CeO_2_ nanoparticles and loading of IBA hormone

#### 2.1.1 Synthesis of Ag@CeO_2_ NPs

The synthesis of Ag@CeO₂ nanoparticles was performed according to the method described by Kayama et al. (2010), with slight modifications. Briefly, 5.049 g (11.628 mmol) of cerium nitrate hexahydrate (Ce(NO₃)₃·6H₂O) and 2.963 g (17.44 mmol) of silver nitrate (AgNO₃) were dissolved in 15 mL of deionized water. To this premixed solution, 3.56 g of ammonia solution (25% NH₄OH) was added dropwise under vigorous stirring at 350 rpm for 1 minute at ambient temperature. The resulting black coprecipitate was subsequently subjected to hydrothermal treatment in an autoclave at 120°C for 10 minutes. The product was then collected by centrifugation, yielding a golden brown solid, which was further calcined at 500°C for 5 hours in air to obtain the final Ag@CeO₂ NPs.

#### 2.1.2 Preparation of IBA−loaded Ag@CeO_2_ NPs

The process of loading IBA onto the Ag@CeO_2_ NPs involved the following steps:

1. Solubilization of IBA:

IBA (60 mg) was dissolved in either 900 μL of absolute ethanol or 900 μL of NaOH solution. The resulting solution was then diluted with 20 mL of an ethanol/water mixture (50:50, v/v) at room temperature to ensure optimal dispersion.

2. Hormone loading:

Subsequently, 60 mg of the previously synthesized Ag@CeO₂ NPs were added to the IBA solution. The mixture was stirred at ambient temperature for 4 hours to facilitate the adsorption of IBA onto the nanoparticle surfaces. After stirring, the solution was allowed to stand undisturbed for 24 hours to maximize loading efficiency.

3. Separation and purification:

The resulting suspension was centrifuged at 10,000 rpm for 15 minutes to separate the IBA–loaded Ag@CeO₂ NPs. The collected nanoparticles were transferred into Eppendorf tubes and stored at −20°C. The frozen nanoparticles were then lyophilized using a freeze-drier (alpha 2–4 LD plus, Christ, Osterode am Harz, Germany).

This process yielded purified IBA–loaded Ag@CeO₂ NPs, which were subsequently characterized and evaluated for their potential applications in enhancing plant development and growth (Karakeçili et al., 2019).

#### 2.1.3 Loading efficiency and controlled release of IBA in PBS medium

The hormone loading efficiency (HLE) and encapsulation efficiency (EE) of IBA in Ag@CeO₂ NPs were evaluated by constructing a standard curve for IBA. To this end, several concentrations of IBA were prepared, and their absorbance was measured using UV-Vis spectroscopy in the wavelength range of 200–800 nm. After establishing the standard curve, a T-test was used to address any issues related to adsorption at specific concentrations. The standard linear equation was then derived, and UV-Vis spectroscopy was employed to measure the absorbance at 221 nm for the IBA-loaded Ag@CeO₂ NPs. The suspension containing IBA-loaded Ag@CeO₂ NPs was centrifuged at 10,000 rpm for 25 minutes at 4°C. The resulting supernatant was diluted twofold, and the samples were analyzed spectroscopically. Using the standard linear equation for IBA and the absorbance at 221 nm, the %EE and %HLE were calculated according to Equations (1) and (2).

Herein, the dialysis bag method was employed to investigate the controlled release of IBA from IBA-loaded Ag@CeO₂ NPs in phosphate-buffered saline (PBS). A cellulose membrane (MWCO: 12 kDa) was used for this study. Initially, 7 mL of the IBA-loaded Ag@CeO₂ NPs suspension was placed into the dialysis bag, which was then submerged in 150 mL of PBS (pH 5.8) at 25°C. Samples were systematically collected at predetermined time intervals (0, 1, 2, 3, 4, 5, 12, 20, 28, 36, 44, 52, 60, and 68 hours) to evaluate the release profile. To maintain a constant total volume, an equivalent amount of PBS was added to the vessel at each sampling point. The release medium volume was kept at least three times greater than the saturation volume of IBA at the experimental temperature (25°C) to ensure sink conditions. All measurements were performed in triplicate. The collected samples were analyzed by UV-Vis spectroscopy at the wavelength corresponding to the maximum absorption of IBA. The resulting data were used to construct a release profile showing the percentage of IBA released from the IBA-loaded Ag@CeO₂ NPs over time. This controlled release study provided important insights into the kinetics and efficacy of IBA release from the nanoparticles in a simulated physiological environment. These findings are essential for understanding the potential applications of IBA-loaded nanoparticles in promoting plant growth and development.

#### 2.1.4 Plant tissue culture experiments

This study investigated tobacco plants (*Nicotiana tabacum*). Plant tissue culture experiments were conducted in a controlled environment. The culture room temperature was maintained at 25°C. Uniform illumination of 1000 lux was provided by fluorescent lamps, following a 16-hour light and 8-hour dark cycle. The Murashige and Skoog (MS) medium, specifically used for plant tissue culture, was prepared according to standard procedures (Murashige and Skoog, 1962). The medium contained 3% sucrose and 0.8% agar, with the pH adjusted to 5.8. All media and equipment were sterilized by autoclaving at 121°C for 15 minutes.

#### 2.1.5 Experimental design and treatments

Stems from the tobacco plant (*Nicotiana tabacum*) were used as the primary experimental material. Stem segments, each approximately 2 cm in length and containing two lateral buds, were excised and cultured in media with different treatments involving IBA–Ag@CeO₂ NPs. The explants were surface sterilized by immersion in 70% ethanol for 1 minute, followed by treatment with 2% sodium hypochlorite solution containing 0.1% Tween-20 for 15 minutes. After sterilization, the explants were rinsed three times with sterile distilled water. The experimental setup included three groups: a control group with hormone-free medium, a group treated with IBA in solution at concentrations of 1, 2, and 3 mgL^-1^, and a group treated with IBA–Ag@CeO₂ NPs at the same concentrations. The stem segments were placed in glass containers measuring 90 mm × 80 mm, each containing 60 mL of culture medium, and maintained under controlled culture conditions.

After four weeks, the stems were evaluated for average root length, maximum root length, fresh weight, dry weight, and time to rooting. The experiment followed a completely randomized design (CRD) with three replications, using two stems per replication. This approach enabled a comparative analysis of the effects of IBA in solution and IBA–loaded nanoparticles on the rooting and growth characteristics of the tobacco stem segments. The control group served as a baseline for assessing the effectiveness of the treatments.

#### 2.1.6 Statistical analysis

The data obtained from the plant tissue culture experiments were analyzed using specific statistical methods. Prior to conducting the ANOVA, the normality of the data distribution was assessed to ensure that the assumptions of ANOVA were met. One-way ANOVA was performed using SPSS version 26 to determine whether significant differences existed among the treatment groups. Following the ANOVA, *post-hoc* Tukey’s HSD tests to compare the mean root length, dry and fresh root weights, and day to rooting among the different concentrations of IBA−Ag@CeO_2_ NPs and Duncan’s Multiple Range Test was employed to compare the means of the different treatment groups and identify any statistically significant differences. The significance level was set at P < 0.05.

## 3 Results

### 3.1 Characterization of the IBA−Ag@CeO_2_ NPs

A comprehensive physicochemical characterization of IBA−Ag@CeO_2_ NPs was performed using multiple techniques, including FT−IR, SEM, UV−visible spectrophotometer, EDAX, XRD, TGA, as well as determination of zeta potential, polydispersity index (PDI), and average particle size.

#### 3.1.1 Assessment of zeta size and zeta potential

**Figure 2:**
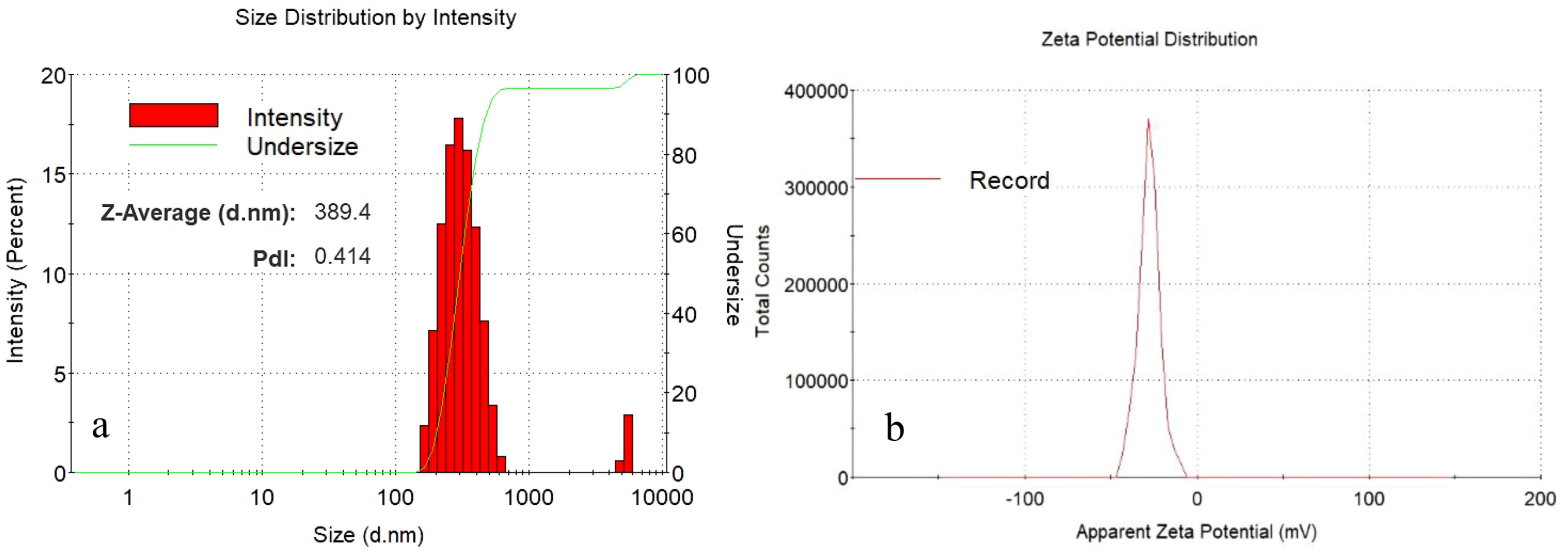
Particle size distribution of **(a)** IBA−Ag@CeO_2_ NPs and zeta potential (mV) value for **(b)** IBA−Ag@CeO_2_ NPs.

ZP and Zetasizer techniques were utilized to characterize IBA−loaded Ag@CeO_2_ NPs (**Figure 2**). After hormone loading, the mean diameter was 364 and 389 nm for Ag@CeO_2_ NPs and synthesized IBA−loaded Ag@CeO_2_ NPs, respectively. PDI=0.414 (<0.5) indicated a colloidal suspension with a uniform particle size distribution. Notably, the increase in hydrodynamic diameter following hormone loading was significant. This variation in particle size can be attributed to particle agglomeration during sample preparation, a phenomenon commonly observed in water-dispersed nanoparticles (Ghaffari et al., 2017). Nanoparticles ranging from 5 to 100 nm can form 200 to 800 nm agglomerates, significantly altering the effective particle size in a fluid (Yang et al., 2012). The zeta potential value of Ag@CeO_2_ NPs is -34.5 mV which exceeds the commonly accepted threshold of ±30 mV for stable nanoparticle suspensions (Berg et al., 2009). This indicates that the synthesized nanoparticles possess good colloidal stability and are unlikely to aggregate under storage and experimental conditions. After hormone loading, the zeta potential decreased to -27.8 mV, showing that the IBA−loaded Ag@CeO_2_ NPs still have good stability.

#### 3.1.2 Electron microscopy analysis

**Figure 3:**
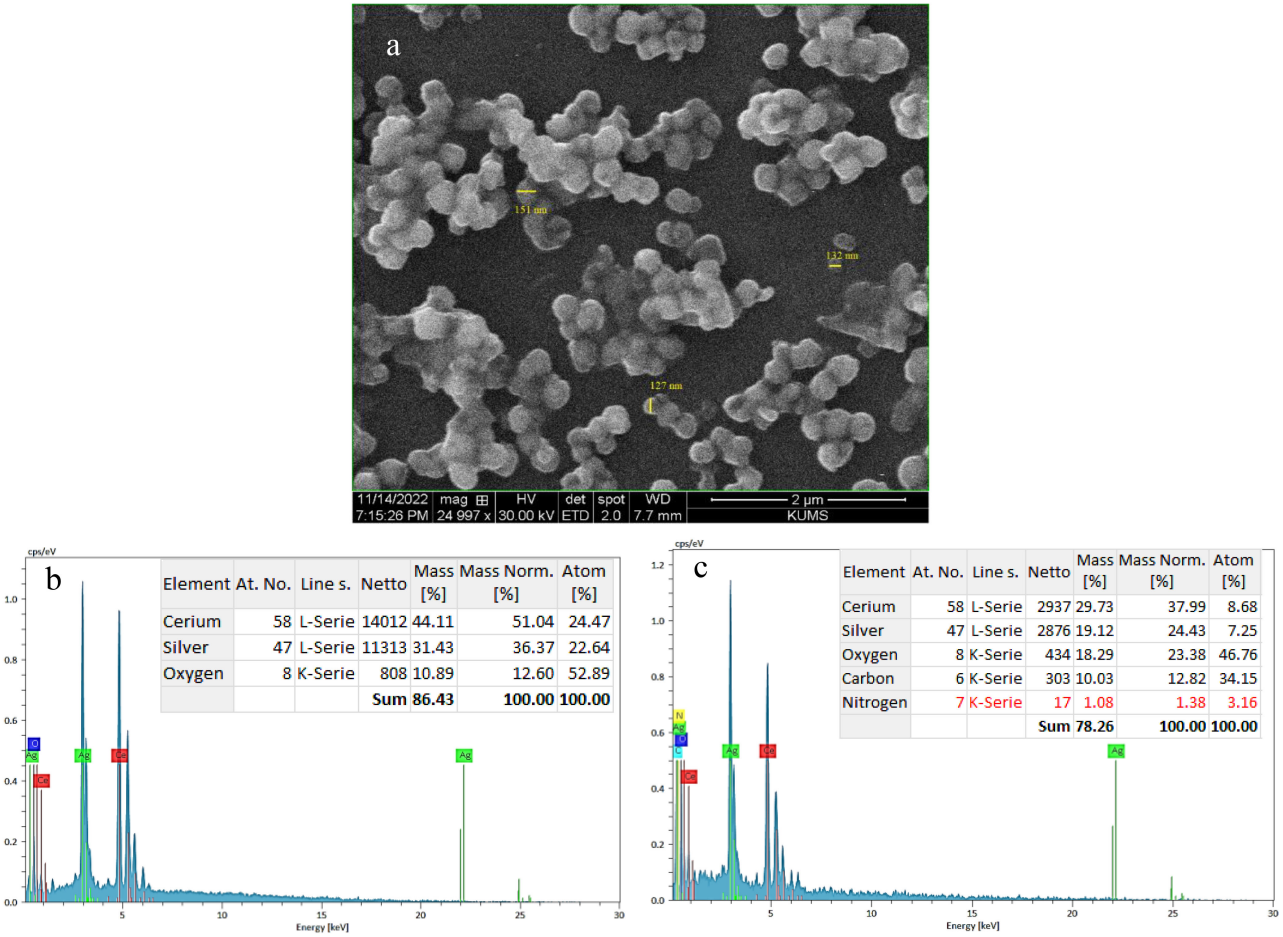
**(a)** SEM image of IBA−Ag@CeO_2_ NPs and EDAX spectra of **(b)** Ag@CeO_2_ NPs and **(c)** IBA−Ag@CeO_2_ NPs.

Elemental characterization and morphological analysis of Ag@CeO_2_ NPs and IBA−Ag@CeO_2_ NPs were performed using a field-emission scanning electron microscope (SEM, Quanta 450, FEI, USA) equipped with an EDAX energy dispersive X-ray analyzer. The SEM image of IBA−Ag@CeO_2_ NPs (**Figure 3A**) revealed a spherical morphology with an average particle size of 136 nm. EDAX analysis (**Figure 3B**) showed that Ag@CeO_2_ NPs contained 51.04% cerium, 36.37% silver, and 12.60% oxygen. After loading with IBA, the intensity of cerium and silver peaks decreased, and a notable carbon content (12.82%) was detected, confirming the presence of IBA molecules (**Figure 3C**). The SEM images indicated that the nanoparticles were uniformly dispersed, with only minor aggregation observed in localized areas. This uniform dispersion suggests successful synthesis and stabilization of the nanoparticles, which is further supported by EDAX elemental mapping confirming the homogeneous distribution of Ag and Ce elements.

#### 3.1.3 Fourier transform infrared analysis

**Figure 4:**
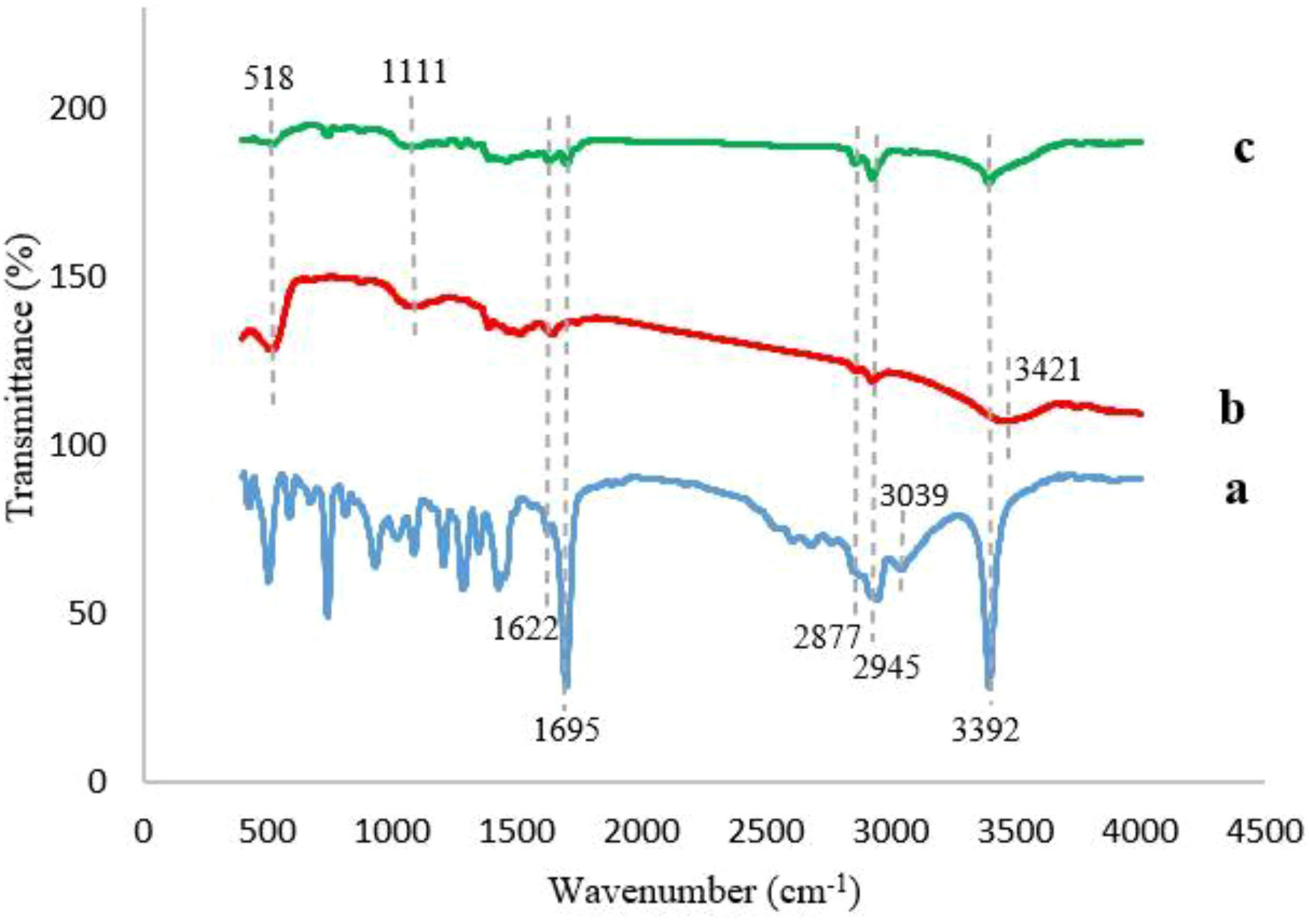
FT−IR Spectra of **(a)** IBA, **(b)** Ag@CeO_2_ NPs, **(c)** IBA−Ag@CeO_2_ NPs.

**Figure 4** illustrates the FTIR spectra of IBA, Ag@CeO_2_ NPs, and IBA−Ag@CeO_2_ NPs. The IBA spectrum (**Figure 4A**) shows distinct peaks corresponding to a variety of molecular vibrations. Particularly, the band at 3392 cm^-1^ is assigned to the stretching vibration of the N–H group. The peaks at 1622, 2877, and 2945 cm^-1^ represent the bending and stretching vibrations of –CH_2_ groups. The vibrations at 1695 cm^-1^ and aromatic C–H stretching vibrations at 3039 cm^-1^ demonstrate the existence of the carboxylate ion (COO^ˉ^) band (Socrates, 1994; Ambrogi et al., 2006). The FT−IR spectrum of Ag@CeO_2_ NPs (**Figure 4B**) observed peaks at 518 cm^-1^ and 1111 cm^-1^ showing in Ce−O bonds (Srivastava et al., 2013). The O−H stretching vibration caused by water adsorption was observed at approximately 3421 cm^-1^. The previous peak was also observed in **Figure 4C**, which was attributed to IBA−Ag@CeO_2_ NPs. The band located at approximately 3390 cm^-1^ arises from the N-H stretching vibration of IBA. The strong peaks observed at 2924 cm^-1^ and 2854 cm^-1^ are also assigned to the C−H stretching vibrations. The presence of the characteristic band at approximately 1699 cm^-1^ is usually associated with the presence of a carbonyl bond (C=O). This observation indicates that the introduction of IBA into the Ag@CeO_2_ NPs is successful.

#### 3.1.4 Hormone loading efficiency and release in PBS

**Figure 5:**
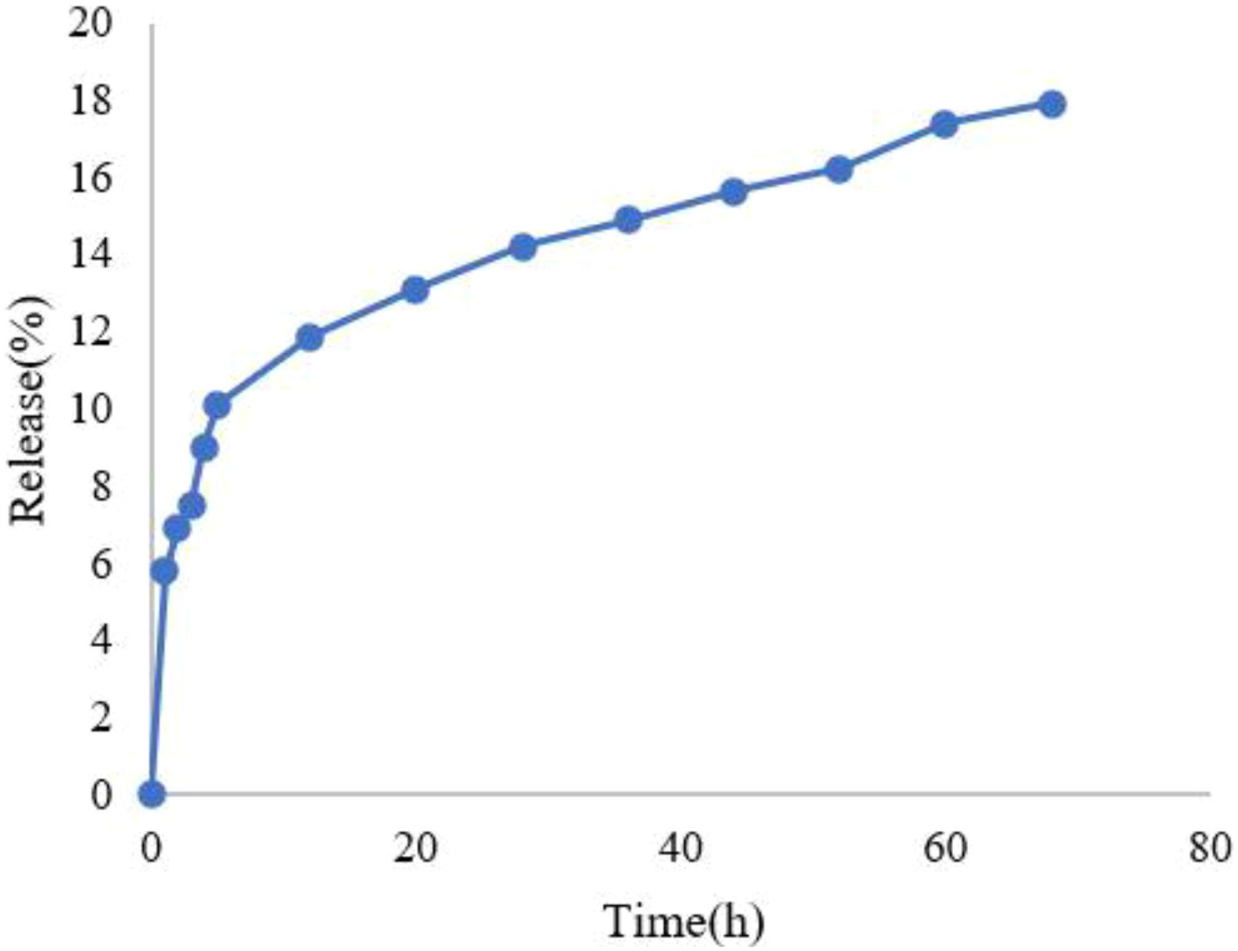
Release diagram of IBA from IBA−loaded Ag@CeO_2_ NPs.

The HLE for IBA was determined to be 45.2 ± 1.8%, while the EE reached 89.7 ± 2.3% (mean ± SD, n=3). Both parameters were calculated based on the standard linear equation derived for IBA. The release profile of IBA from IBA−Ag@CeO_2_ NPs was evaluated using a dialysis bag assay and UV spectroscopy. Results showed an initial rapid release of 15% within the first 36 hours, followed by a sustained and steady release, reaching a cumulative total of 18% over 68 hours (**Figure 5**).

In this study, the release of IBA from Ag@CeO_2_ NPs followed the zero-order kinetic model, considering the correlation coefficient. In zero-order kinetics, the release rate of the active ingredient remains constant over time and is independent of the concentration remaining in the carrier. This model is increasingly applied in agricultural nanoparticle systems because it ensures a consistent delivery rate, which is critical for optimizing plant uptake and reducing environmental impact (Haydar et al., 2024).

#### 3.1.5 XRD analysis

**Figure 6:**
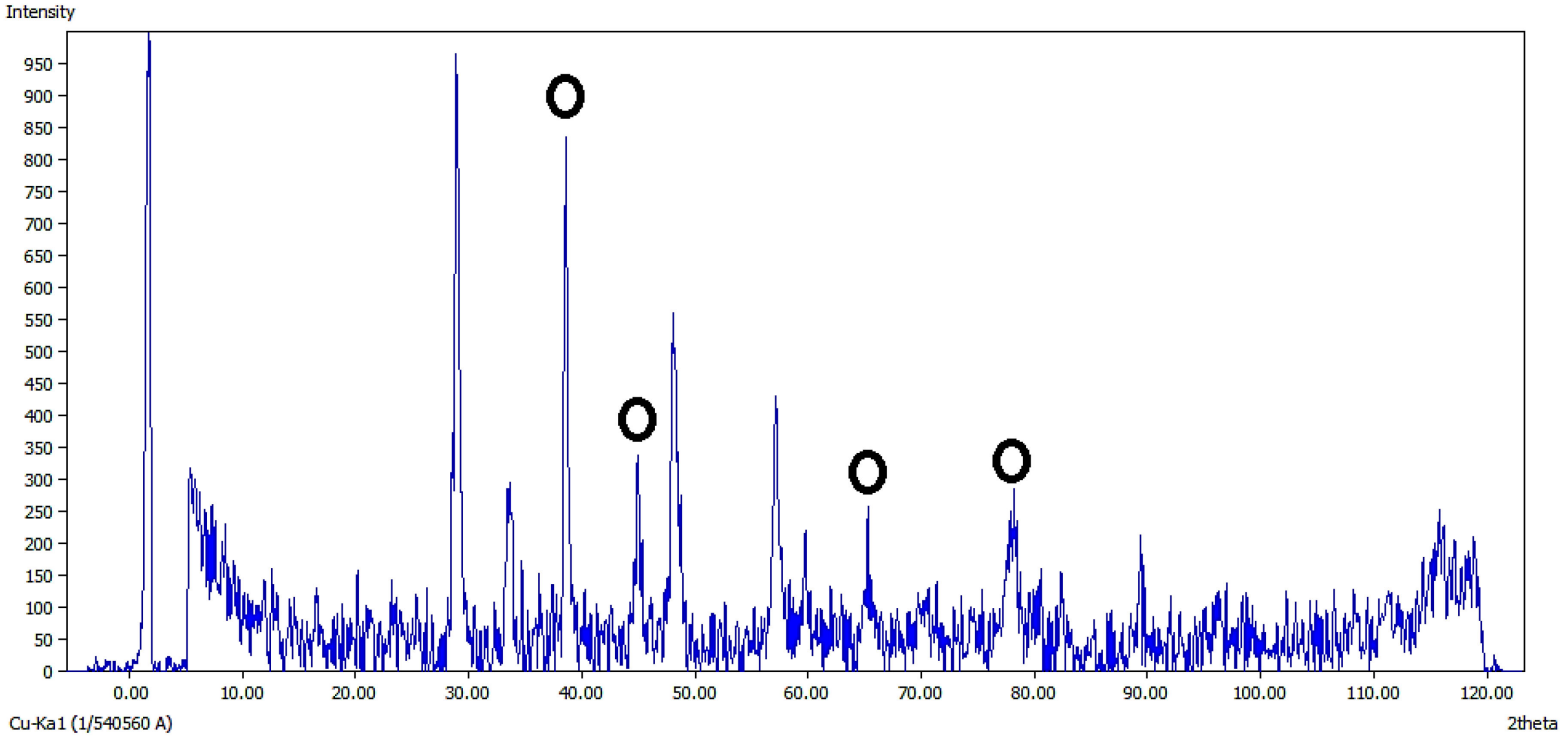
XRD pattern of Ag@CeO_2_NPs.

The XRD spectrum of Ag@CeO₂ NPs, measured using Cu Kα radiation (**Figure 6**), confirms the crystalline structure and phase composition. Distinct peaks at 2θ = 28.6°, 33.2°, 47.5°, 56.5°, 59.2°, 69.4°, 76.7°, and 79.1°correspond to the (111), (200), (220), (311), (222), (400), (331), and (420) crystal planes of the face-centered cubic (FCC) phase of CeO₂. These peaks align with the standard cubic fluorite structure of CeO₂ (JCPDF 75-8371) (Palard et al., 2010). Additional peaks attributed to metallic Ag are observed, confirming the coexistence of Ag and CeO₂ phases. The absence of peak shifts in the CeO₂ diffraction pattern suggests that Ag nanoparticles are surface-decorated rather than incorporated into the CeO₂ lattice. The sharp and well-defined peaks indicate high crystallinity, while the distinct separation between Ag and CeO₂ reflections demonstrates the successful synthesis of Ag@CeO₂ nanocomposites.

#### 3.1.6 Thermal analysis

**Figure 7:**
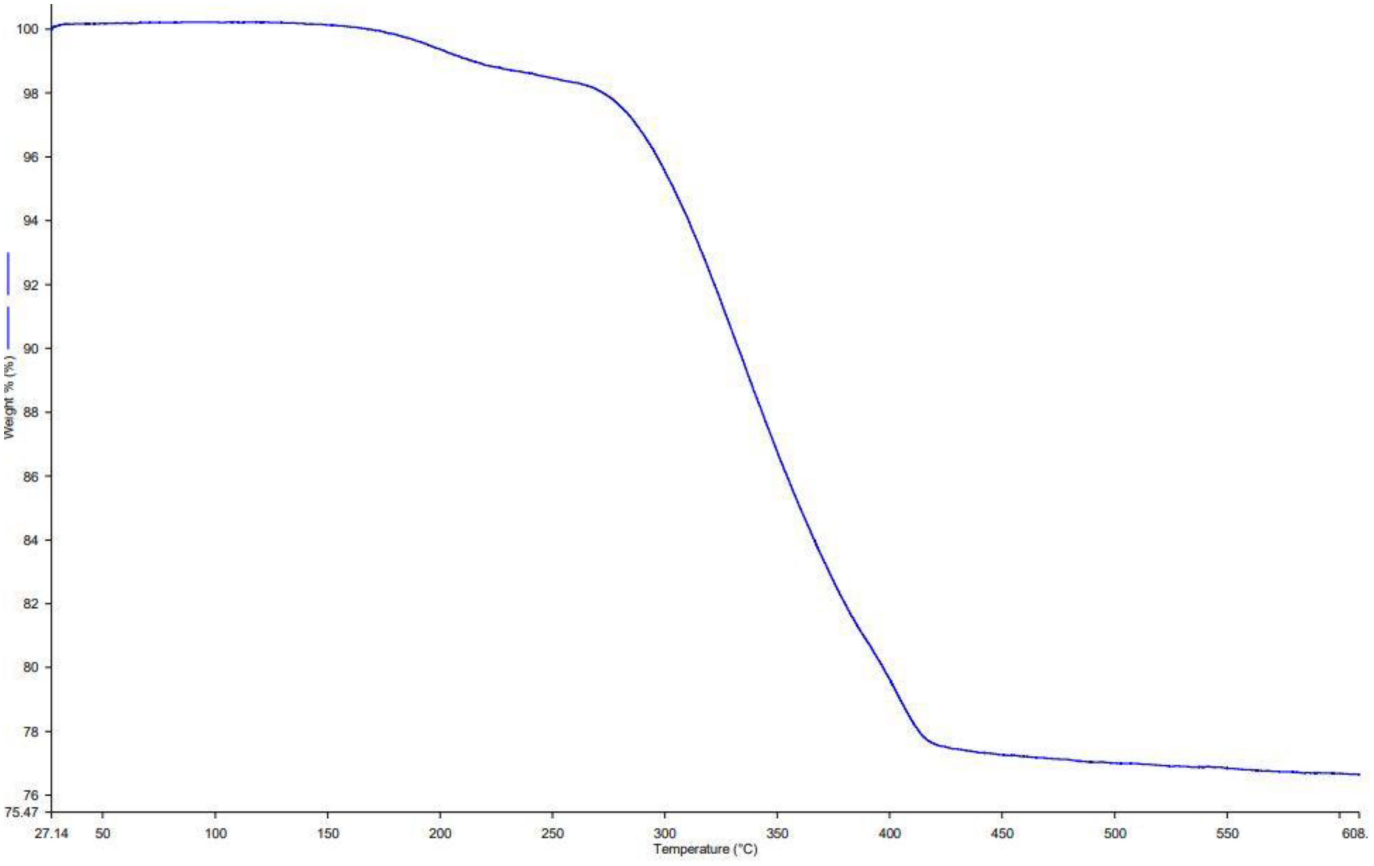
TGA curve of IBA−Ag@CeO_2_ NPs.

To evaluate the IBA content and thermal stability of Ag@CeO_2_ NPs, thermogravimetric analysis (TGA) was performed (**Figure 7**). The TGA curve of IBA-loaded Ag@CeO_2_ NPs displayed three distinct weight loss steps. The first step, accounting for 3.2% weight loss below 150 °C, corresponds to the loss of adsorbed water. The second stage, with a 12.8% weight loss between 150–350 °C, is attributed to the decomposition of organic residues and the loaded IBA. The final stage, occurring above 350°C, resulted in an additional 4.5% weight loss, which is likely due to further degradation and stabilization of the nanoparticle structure. These results confirm the successful loading of IBA onto Ag@CeO_2_ NPs and demonstrate the thermal stability of the nanocomposite across a broad temperature range.

#### 3.1.7 The effect of IBA−Ag@CeO_2_ NPs on *Nicotiana tabacum* Rooting

**Figure 8:**
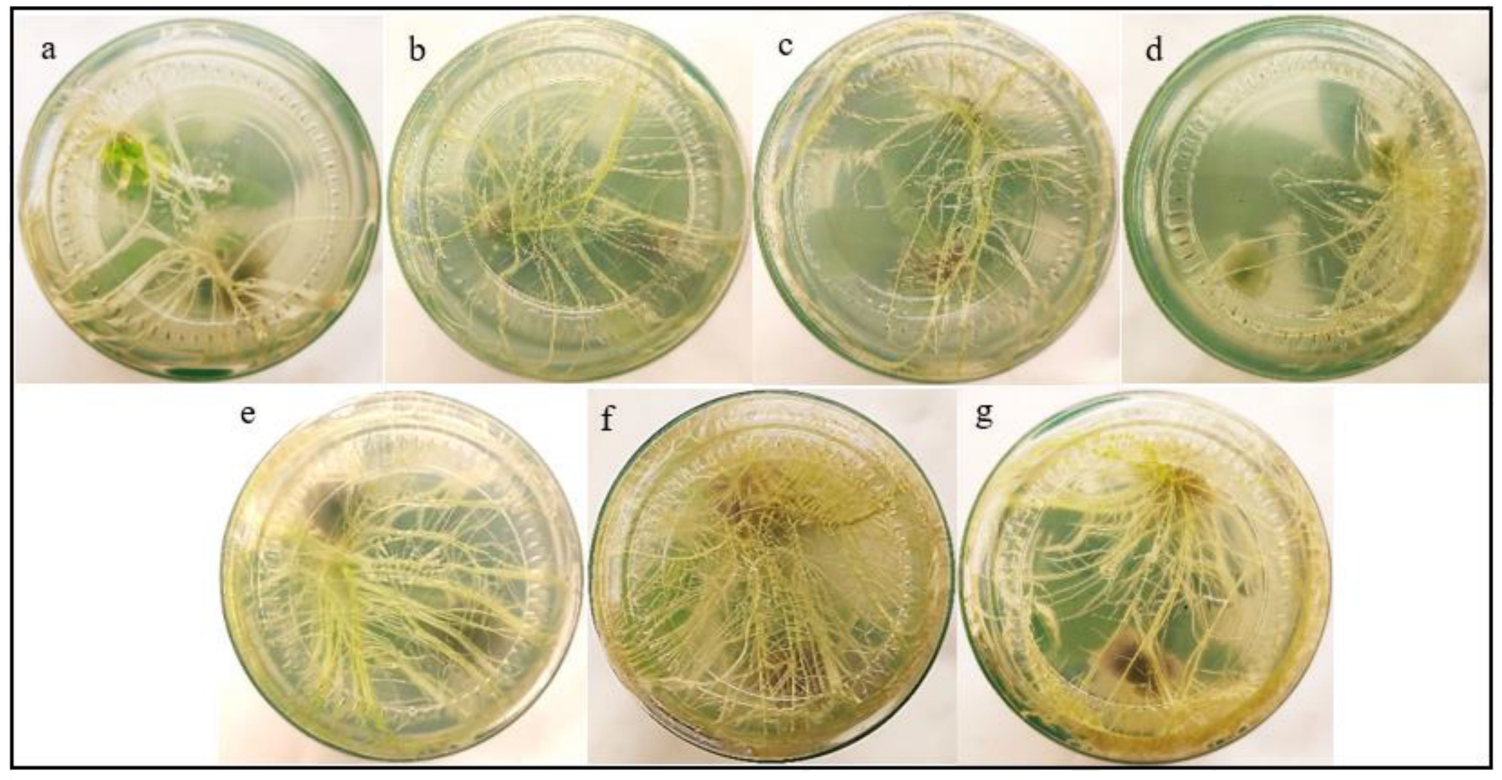
Effects of free IBA, and IBA-Ag@CeO_2_ NPs on *in vitro* rooting of tobacco plant stem. **(a)** Control 0.0 mg L^–1^ IBA, **(b)** 1.0 mg L^–1^ IBA, **(c)** 2.0 mg ^L–1^ IBA, **(d)** 3.0 mg ^L–1^ IBA, **(e)** IBA-Ag@CeO_2_ NPs (1.0 mg L^–1^ IBA), **(f)** IBA-Ag@CeO_2_ NPs (2.0 mg ^L–1^ IBA), **(g)** IBA-Ag@CeO_2_ NPs (3.0 mg ^L–1^ IBA). Figure f clearly shows that the 2 mgL^-1^ IBA-Ag@CeO_2_ NP treatment significantly outperformed other concentrations in development root growth in tobacco plants.

To our knowledge, this is the first study to explore the use of IBA−Ag@CeO_2_ NPs for the delivery of auxin to improve rooting in tobacco. **Figure 8** and **Table 1** present the impacts of IBA and IBA−Ag@CeO_2_ NPs on mean root length, length of the longest roots, days to rooting, and root fresh/dry weight. To rigorously determine the optimal concentration of IBA-Ag@CeO_2_ NPs, we performed a one-way ANOVA followed by a *post-hoc* Tukey’s HSD test to compare the effects of different concentrations (1, 2, and 3 mg L^−1^) on root length, root fresh weight, root dry weight, and day to rooting. Among the root length attributes, the highest root length value of the IBA−Ag@CeO_2_ NPs treatment group with a concentration of 2 mgL^-1^ was 9.66 cm, and the lowest root length value of the control group was 5.60 cm. The longest root length belongs to the IBA−Ag@CeO_2_ NPs treatment with a concentration of 2 mgL^-1^ and a value of 10.03 cm. Moreover, under the same treatment and the same concentration, the root fresh weight and root dry weight characteristics were the highest, 0.78 g and 0.05 g, respectively, which were statistically significantly different from other treatments. In addition, we observed that the fastest rooting time among different treatments was the IBA−Ag@CeO_2_ NPs treatment with a concentration of 2 mgL^-1^ with, a rooting speed of 2 days, which was statistically significantly different from other treatments. The results of one-way ANOVA revealed significant differences among treatments for mean root length (F(3, 12) = 15.42, p = 0.001), fresh root weight (F(3, 12) = 10.27, p = 0.002), and days to rooting (F(3, 12) = 8.95, p = 0.004). *Post hoc* analysis using Tukey’s HSD test indicated that the 2 mgL^-1^ IBA−Ag@CeO_2_ NPs treatment resulted in significantly higher root length and fresh weight compared to the control group and other concentrations (p < 0.05). Additionally, the days to rooting were significantly reduced in the 2 mgL^-1^ treatment group compared to the control and other concentrations (p < 0.05). Our results suggest that Ag@CeO_2_ NPs function as an effective nanocarrier for delivering the IBA hormone, highlighting their potential applications in agricultural practices.

**Table 1:** The effect of hormone indole−3−butyric acid loaded on silver−ceria nanoparticles (IBA−Ag@CeO_2_ NPs) on the rooting of tobacco plants *in*−*vitro*.

Treatment	Auxin concentration (mg L-1)	Root length (cm)	Longest root length (cm)	Fresh weight (g)	Dry weight (g)	Day to rooting (day)
Control	0.0	5.60 ± 0.15 d	6.10 ± 0.20 d	0.04 ± 0.005 e	0.03 ± 0.002 bc	9.33 ± 0.50 b
IBA	1.0	7.50 ± 0.20 a	7.80 ± 0.25 b	0.40 ± 0.05 cd	0.02 ± 0.002 c	12.33 ± 0.60 a
	2.0	7.66 ± 0.22 a	8.00 ± 0.30 b	0.53 ± 0.06 bc	0.03 ± 0.003 bc	9.33 ± 0.50 b
	3.0	6.40 ± 0.18 c	6.70 ± 0.22 c	0.33 ± 0.04 d	0.02 ± 0.002 c	12.66 ± 0.65 a
IBA−Ag@CeO_2_	1.0	8.90 ± 0.28 a	9.76 ± 0.32 a	0.62 ± 0.06 b	0.04 ± 0.004 b	5.66 ± 0.40 d
	2.0	9.66 ± 0.31 a	10.03 ± 038 a	0.78 ± 0.05 a	0.05 ± 0.004 a	2.00 ± 0.30 e
	3.0	7.36 ± 0.21 b	7.90 ± 0.26 b	0.53 ± 0.04 bc	0.03 ± 0.003 bc	7.66 ± 0.45 c

Different letters within each column indicate statistical differences at P < 0.05.

Effect of different concentrations of IBA−Ag@CeO_2_ NPs on rooting of tobacco plants. Data represent mean ± SD (n=3). One-way ANOVA showed significant differences among treatments (F(3,12) = 15.42, p = 0.001). *Post hoc* Tukey’s HSD test indicated that the 2 mgL^-1^ treatment group had significantly longer roots compared to control, 1 mgL^-1^, and 3 mgL^-1^ groups (p < 0.05).

## 4 Discussion

### 4.1 Nanoparticle characterization

From the perspective of nanobiotechnology, metal oxide nanoparticle Ag@CeO_2_ are suitable carrier for IBA hormone transfer. This causes the plant roots to grow rapidly. Its physical and chemical properties were confirmed through different analyses. In a study conducted by McMillan et al. (2011) the size and shape of nanoparticles affects the way they interact with cells, thereby determining their distribution pattern, toxicity, and ability to target cells. Studies show that smaller nanoparticles (around 100 nm) have significantly higher cellular uptake and absorptive capacity compared to larger particles, which enhances their ability to deliver active compounds into plant tissues (Desai et al., 1997). Therefore, Ag@CeO_2_ NPs are expected to have the ability to interact with plant cells. In addition, our results demonstrated that the reduction in negative charge observed in the zeta potential test may be attributed to the coating of this nanostructure with IBA molecules, which suggests the stability of our structure. The zeta potential can influence the interaction of nanoparticles with plant cells, including mechanisms of attachment and uptake. A lower negative charge can promote cellular uptake, potentially enhancing the delivery of IBA to plant tissues and facilitating improved root development. Negatively charged CeO_2_ NPs are more efficiently translocated within plants, while positively charged NPs tend to adhere to root surfaces. These nanoparticles can sustain hormone levels for an extended duration due to their colloidal stability. Additionally, they can function as nanofertilizers, amplifying the effects of plant hormones. The stability of nanoparticles and their biological applications are significantly influenced by their interactions with biological systems. Recent advancements in the use of biosurfactants for stabilizing metallic nanoparticles have shown promise due to their ability to prevent agglomeration (Femina Carolin and Kamalesh, 2024). Furthermore, the conjugation of biomolecules with nanoparticles has proven effective in maintaining stability while enhancing biocompatibility and functionality in biological environments (Lee et al., 2021).

In the study by Korpayev et al. (2021), the SEM images showed spherical and monodisperse IBA−loaded nAg nanoparticles with a size of approximately 100 nm. Which confirms our results. The hydrodynamic size of the nanoparticles is larger compared to the sizes obtained from the SEM images in the sample. The results of the two analyses are not directly comparable. Because the hydrodynamic diameter in a colloidal aqueous solution includes the solute layer (water molecules and ions). It is also possible to form small clusters in the solution state, which results in an increase in the size of the hydrodynamic diameter in DLS testing compared to the size of the nanoparticles in SEM testing.

In this study, we found that a high encapsulation efficiency of ~90% suggests that a significant portion of the IBA was effectively incorporated into the nanoparticles during the loading process. The HLE of 45% indicates that a substantial amount of the loaded IBA was retained within the nanoparticles, making it available for potential release and uptake by plant tissues. Also, the release rate of IBA from Ag@CeO₂ NPs was 18% in 68 hours, indicating different biological effects. IBA is metabolized to IAA, which is essential for various growth processes. A low release may result in insufficient IAA levels, limiting growth responses such as cell expansion and root development (Strader et al., 2010). The slow release could affect the absorption rates in plant tissues, potentially leading to suboptimal physiological responses (Ludwig-Müller, 2000). Conversely, while low IBA release may limit its immediate effects, it could also suggest a controlled release mechanism that prevents excessive auxin levels, which can lead to negative physiological effects in plants. This balance may be crucial for maintaining optimal growth conditions. Importantly, no callus formation was noted across all treatments, including those with IBA or IBA-loaded Ag@CeO₂ NPs, as well as in the control group. This absence of callus formation is advantageous, as it supports quality root development in tobacco plants. The lack of callus formation may be attributed to the optimal concentrations of IBA−loaded Ag@CeO₂ NPs utilized in the plant cells. We observed that EDAX analysis showed the presence of 12.82% carbon in the IBA−Ag@CeO_2_ NPs. Moreover, the study by Korpayev et al. (2021) showed that 12.48% carbon and 1.96% nitrogen were detected in IBA−nAg particles. These results confirm the presence of IBA hormone on the nanoparticles. Also, the production of Ag@CeO_2_ NPs was confirmed by XRD analysis and the presence of IBA on Ag@CeO_2_ NPs was confirmed by FT−IR analysis.

The results of a study conducted by Thangavelu et al. (2018) showed that the decomposition and denaturation rate of IBA hormone in silver nanoparticles starts at 100°C and is completely experienced at 300°C. Therefore, IBA loaded on Ag@CeO_2_ NPs synthesized in our study, which was stable up to 420°C, showed that this nanostructure has better thermal stability compared to silver nanoparticles. Thermal stability ensures that nanoparticles maintain their structure and function, allowing for a controlled and sustained release of plant hormones, which is critical for effective plant growth and development (Gohar et al., 2024). Moreover, environmental stressors such as temperature variations are common in agricultural settings. This resilience helps in maintaining the efficacy of hormone delivery systems. Stable nanoparticles facilitate better nutrient and hormone uptake, enhancing plant growth and productivity. This is particularly important in nutrient-deficient soils where efficient delivery systems can make a significant difference (Sharma et al., 2024).

### 4.2 The effectiveness of nanoparticles in plant growth

Our results demonstrated that among different concentrations of nanoparticles, IBA−Ag@CeO_2_ NPs at a concentration of 2 mgL^-1^ had more effective and promising results than other treatments in promoting root growth. These findings underscore the potential of Ag@CeO_2_ NPs as an efficient delivery system for the IBA hormone in plant tissue culture and micropropagation applications. The comprehensive physicochemical characterization confirms the suitability and stability of the nanoparticles as a carrier system, ensuring that a significant amount of the active IBA hormone can be effectively delivered to plant tissues. Also, our previous studies related to the effect of hollow mesoporous silica nanoparticles and alginate-chitosan nanocapsules with IBA hormone showed that these carriers cause the stability of this hormone, the controlled release of IBA from these nanocarriers and increase the growth of tobacco plant roots (Ghorbani et al., 2024a; Ghorbani et al., 2024b). Research in other crops supports these observations. To increase the rooting potential of guava cuttings (*Psidium guajava* L), a study was conducted by Abdallatif et al. (2022) to investigate the effect of 1−Naphthaleneatic acid (NAA) and IBA alone and together with silver nanoparticles on the rooting of guava cuttings. Treatments with silver nanoparticles improved rooting efficiency compared to auxin alone. NAA−loaded silver nanoparticles treatment developed a lower number of long roots, while IBA−loaded silver nanoparticles recorded higher values of all root parameters. Thangavelu et al. (2018) demonstrated the successful synthesis of Ag NPs using two rooting hormones (auxin indole−3−acetic acid (IAA) and IBA) as stabilizers. The effectiveness of its rooting and pathogen inhibiting properties was also fully verified through *in vitro* and ex vivo studies involving model plants and plant pathogens. The hormone-containing silver nanoparticles showed a dual effect. In addition to inhibiting the pathogen, they increased root growth threefold compared to the control group.

A recent investigation conducted by Korpayev et al. (2021) revealed that the application of chitosan and silver nanoparticles loaded with IAA or IBA, resulted in a substantial enhancement of *in vitro* adventitious root formation in Malling Merton 106 (MM 106) micro-cuttings, with increases ranging from 33.3% to 50.0% when compared to the use of free IAA or IBA. Research by Karakeçili et al. (2019) indicated that using IBA−loaded zinc oxide (ZnO) or IAA NPs increased *in vitro* rooting efficiency twofold for the difficult-to-root wild pear (*Pyrus elaeagrifolia Pallas*). In that study, the highest rooting percentage was achieved with IBA−loaded ZnO NPs and IAA−loaded ZnO NPs at a concentration of 400 mgL^-1^, resulting in rooting percentages of 50.0% and 41.7% respectively. Additionally, Reed et al. (2013) conducted a similar study on various Pyrus genotypes, focusing on auxins’ effects on rooting in challenging genotypes. They found that conventional auxin application resulted in a maximum rooting percentage of only 31.8%. Therefore, our results indicate that Ag@CeO_2_ NPs have a positive effect on the transfer of IBA hormone to improve the rooting index of tobacco plants.

Based on tissue culture results of tobacco plants treated with Ag@CeO_2_ NPs, we observed increased root growth compared to the control group. Moreover, the samples continued to grow well during the growth phase, which actually indicates that our nanoparticles are not cytotoxic. Furthermore, the concentration of nanoparticles used in this study was chosen based on the study conducted by Korpayev et al. (2021), who did not report toxicity of silver nanoparticles at these concentrations. However, long−term exposure to Ag−NPs alters microbial diversity, with root treatments leading to reduced bacterial and fungal biodiversity (Vitali et al., 2019). Studies show that Ag−CeO_2_ NPs can inhibit growth in various plant species, with effects varying by concentration of nanoparticle (Viswanathan et al., 2024). The antibacterial properties of silver may inadvertently disrupt human microbiota, potentially leading to immune system imbalances (Mahapatra et al., 2024). The accumulation of Ag−CeO_2_ NPs in the food chain can lead to human exposure, potentially impacting immune responses due to their toxic properties (Grün et al., 2018). In contrast, some studies suggest that nanoparticles can enhance certain agricultural practices by improving crop yields in the short term. However, the long-term ecological and health implications of their accumulation in the environment necessitate careful consideration and further research.

## 5 Conclusion

Ag@CeO₂ NPs have proven to be effective carriers for the delivery of IBA and for enhancing rooting in tobacco plants. Their physicochemical properties have been thoroughly validated through various analytical methods, including SEM, EDAX, FTIR, XRD, and TGA. Given a high loading capacity of 90% for IBA, these nanoparticles demonstrate their ability to effectively encapsulate and deliver the active compound. The application of IBA−Ag@CeO₂ NPs led to a notable increase in root length and fresh/dry weight compared to control groups and other treatments, highlighting the effectiveness of this nanocarrier system. The controlled release profile of IBA from the nanoparticles followed a zero−order kinetic model, which is beneficial for maintaining optimal hormone levels in plant tissues, thus preventing excessive auxin levels that could negatively impact plant physiology. The study identified an optimal concentration of 2 mg L^−1^ of IBA−loaded Ag@CeO₂ NPs, which yielded more effective results in promoting root growth compared to other treatments. Whereas this research provided valuable insights, its limitations and the need for further research should also be acknowledged. Future investigations are recommended to aim at optimizing nanoparticle formulations to enhance their effectiveness, assessing their applicability across diverse plant species, and examining various plant growth regulators (PGRs) and nanoparticle systems. A limitation of the present study is the absence of a nanoparticle-only control (Ag@CeO₂ without IBA) in our experimental design. This limitation is important to acknowledge as recent studies have demonstrated that metal oxide nanoparticles, particularly cerium oxide nanoparticles, can influence plant growth and development (Li et al., 2024). Future research should incorporate a nanoparticle-only control group to better distinguish between the effects of the nanocarrier itself and those of the hormone. This would provide a more comprehensive understanding of the mechanisms underlying the enhanced rooting observed in this study and further optimize nanocarrier−based hormone delivery systems for plant biotechnology applications. This research has significant practical implications, as it has the potential to improve plant productivity and growth in agriculture. The developed IBA-loaded Ag@CeO₂ nanoparticle system offers a promising approach for farmers, horticulturists and commercial nurseries to enhance root development in economically important plants. By providing controlled hormone release, with potentially reduced environmental impact compared to conventional methods, this technology could contribute to more efficient plant propagation protocols, improved transplant success rates, and ultimately enhanced crop productivity in various crop systems.

## Data Availability

The raw data supporting the conclusions of this article will be made available by the authors, without undue reservation.

## References

[B1] Abdallatif A. M. Hmmam. I. Ali M. A. (2022). Impact of silver nanoparticles mixture with NAA and IBA on rooting potential of Psidium guajava L. stem cuttings. Egypt. J. Chem. 65, 1119–1128. doi: 10.21608/ejchem.2022.155986.6751

[B2] Abdi G. Salehi H. Khosh-Khui M. (2008). Nano silver: a novel nanomaterial for removal of bacterial contaminants in valerian (Valeriana officinalis L.) tissue culture. Acta Physiol. Plant 30, 709–714. doi: 10.1007/s11738-008-0169-z

[B3] Aghdaei M. Sarmast M. Salehi H. (2012). Effects of silver nanoparticles on Tecomella undulata (Roxb.) Seem. micropropagation. Advances in horticultural science 21–24. doi: 10.13128/ahs-12748

[B4] Ambrogi V. Famiani F. Perioli L. Marmottini F. Di Cunzolo I. Rossi C. (2006). Effect of MCM-41 on the dissolution rate of the poorly soluble plant growth regulator, the indole-3-butyric acid. Microporous Mesoporous Mater. 96, 177–183. doi: 10.1016/j.micromeso.2006.06.033

[B5] Amiri H. Mousavi M. Torahi A. (2016). Improving date palm (phoenix dactylifera L. cv. estamaran) calogenesis by the use of zinc oxide nanoparticles. J. Exp. Biol. Agric. Sci. 4, 557–563. doi: 10.18006/2016.4(5).557.563

[B6] Berg J. M. Romoser A. Banerjee N. Zebda R. Sayes C. M. (2009). The relationship between pH and zeta potential of∼ 30 nm metal oxide nanoparticle suspensions relevant to *in vitro* toxicological evaluations. Nanotoxicology 3, 276–283. doi: 10.3109/17435390903276941

[B7] Celardo I. Pedersen J. Z. Traversa E. Ghibelli L. (2011). Pharmacological potential of cerium oxide nanoparticles. Nanoscale 3, 1411–1420. doi: 10.1039/C0NR00875C 21369578

[B8] Chutipaijit S. (2015). Establishment of condition and nanoparticle factors influencing plant regeneration from aromatic rice (Oryza sativa). Int. J. Agric. Biol. 17, 1049–1054. doi: 10.17957/ijab/15.0030

[B9] Clemente I. Falsini S. Di Cola E. Fadda G. C. Gonnelli C. Spinozzi F.. (2019). Green nanovectors for phytodrug delivery: in-depth structural and morphological characterization. ACS Sustain. Chem. Eng 7, 12838–12846. doi: 10.1021/acssuschemeng.9b01748

[B10] Conesa J. (1995). Computer modeling of surfaces and defects on cerium dioxide. Surface Science. 339, 337–352. doi: 10.1016/0039-6028(95)00595-1

[B11] Damodaran S. Strader L. C. (2019). Indole 3-butyric acid metabolism and transport in Arabidopsis thaliana. Front. Plant Sci. 10. doi: 10.3389/fpls.2019.00851 PMC6616111 31333697

[B12] Desai M. P. Labhasetwar V. Walter E. Levy R. J. Amidon G. L. (1997). The mechanism of uptake of biodegradable microparticles in Caco-2 cells is size dependent. Pharm. Res. 14, 1568–1573. doi: 10.1023/A:1012126301290 9434276

[B13] Dong H. Guo M. Liang Y. Fan C. Ding G. Zhang W.. (2018). Preparation and characterization of indole-3-butyric acid nanospheres for improving its stability and utilization. Mater. Sci. Eng. C. 89, 175–181. doi: 10.1016/j.msec.2018.04.004 29752087

[B14] Epstein E. Ludwig-Müller J. (1993). Indole-3-butyric acid in plants: occurrence, synthesis, metabolism and transport. Physiol. Plant 88, 382–389. doi: 10.1111/j.1399-3054.1993.tb05513.x

[B15] Femina Carolin C. Kamalesh T. (2024). Advances in stabilization of metallic nanoparticle with biosurfactants-A review on current trends. Heliyon 10, e29773. doi: 10.1016/j.heliyon.2024.e29773 38699002 PMC11064090

[B16] Ghaffari S. B. Sarrafzadeh M. H. Fakhroueian Z. Shahriari S. Khorramizadeh M. R. (2017). Functionalization of ZnO nanoparticles by 3-mercaptopropionic acid for aqueous curcumin delivery: Synthesis, characterization, and anticancer assessment. Mater. Sci. Eng. C. 79, 465–472. doi: 10.1016/j.msec.2017.05.065 28629042

[B17] Ghorbani M. Kahrizi D. (2024). Innovative capsulation and microencapsulation of plant hormones: a strategy to combat plant pathogens. Cell. Mol. Biol. 70, 10–17. doi: 10.14715/cmb/2024.70.12.2 39799502

[B18] Ghorbani M. Kahrizi D. Arkan E. Aghaz F. Zebarjadi A. (2024b). Enhancing rooting tobacco (Nicotiana tabacum) plant by loaded indole-3-butyric acid in alginate/chitosan nanocapsule. Cell. Mol. Biol. 70, 237–242. doi: 10.14715/cmb/2024.70.7.34 39097868

[B19] Ghorbani M. Kahrizi D. Arkan E. Aghaz F. Zebarjadi A. Ghorbani S. (2024a). Synthesis and characterization of indole-3-butyric acid-loaded hollow mesoporous silica nanoparticles: effects on plant rooting induction. J. Plant Growth Regul. 43, 4506–4516. doi: 10.1007/s00344-024-11411-x

[B20] Gohar F. Iqbal U. Z. Khan M. B. Rehman F. Maryam F. S. U. Azmat M.. (2024). Impact of nanoparticles on plant growth, development and physiological processes: A comprehensive review. doi: 10.20944/preprints202410.0780.v1

[B21] Grün A. L. Straskraba S. Schulz S. Schloter M. Emmerling C. (2018). Long-term effects of environmentally relevant concentrations of silver nanoparticles on microbial biomass, enzyme activity, and functional genes involved in the nitrogen cycle of loamy soil. J. Environ. Sci. 69, 12–22. doi: 10.1016/j.jes.2018.04.013 29941247

[B22] Haydar M. S. Ghosh D. Roy S. (2024). Slow and controlled release nanofertilizers as an efficient tool for sustainable agriculture: Recent understanding and concerns. Plant Nano Biol. 7, 100058. doi: 10.1016/j.plana.2024.100058

[B23] Kandasamy G. Maity D. (2015). Recent advances in superparamagnetic iron oxide nanoparticles (SPIONs) for *in vitro* and *in vivo* cancer nanotheranostics. J. Plant Sci. 496, 191–218. doi: 10.1016/j.ijpharm.2015.10.058 26520409

[B24] Karakeçili A. Korpayev S. Dumanoğlu H. Alizadeh S. (2019). Synthesis of indole-3-acetic acid and indole-3-butyric acid loaded zinc oxide nanoparticles: Effects on rhizogenesis. J. Biotechnol. 303, 8–15. doi: 10.1016/j.jbiotec.2019.07.004 31301312

[B25] Karakoti A. S. Tsigkou O. Yue S. Lee P. D. Stevens M. M. Jones J. R.. (2010). Rare earth oxides as nanoadditives in 3-D nanocomposite scaffolds for bone regeneration. J. Mate. Chem. 20, 8912–8919. doi: 10.1039/C0JM01072C

[B26] Kashyap P. L. Xiang X. Heiden P. (2015). Chitosan nanoparticle based delivery systems for sustainable agriculture. Int. J. Biol. Macromol. 77, 36–51. doi: 10.1016/j.ijbiomac.2015.02.039 25748851

[B27] Kayama T. Yamazaki K. Shinjoh H. (2010). Nanostructured ceria– silver synthesized in a one-pot redox reaction catalyzes carbon oxidation. J. Am. Chem. Soc 132, 13154–13155. doi: 10.1021/ja105403x 20825187

[B28] Korpayev S. Karakeçili A. Dumanoğlu H. Ibrahim Ahmed Osman S. (2021). Chitosan and silver nanoparticles are attractive auxin carriers: A comparative study on the adventitious rooting of microcuttings in apple rootstocks. Biotechnol. J. 16, 2100046. doi: 10.1002/biot.202100046 34028191

[B29] Kumari R. Singh D. P. (2016). “Silver nanoparticle in agroecosystem: Applicability on plant and risk-benefit assessment,” in Plant responses to xenobiotics, Springer, Singapore. 293–305. doi: 10.1007/978-981-10-2860-1_12

[B30] Labeeuw L. Khey J. Bramucci A. R. Atwal H. de la Mata A. P. Harynuk J.. (2016). Indole-3-acetic acid is produced by Emiliania huxleyi coccolith-bearing cells and triggers a physiological response in bald cells. Front. Microbiol. 7. doi: 10.3389/fmicb.2016.00828 PMC4896954 27375567

[B31] Lee J. W. Choi S. R. Heo J. H. (2021). Simultaneous stabilization and functionalization of gold nanoparticles via biomolecule conjugation: Progress and perspectives. ACS Appl. Mater. Interfaces 13, 42311–42328. doi: 10.1021/acsami.1c10436 34464527

[B32] Li G. Gao Q. Nyande A. Dong Z. Khan E. H. Han Y.. (2024). Cerium oxide nanoparticles promoted lateral root formation in Arabidopsis by modulating reactive oxygen species and Ca2+ level. Funct. Plant Biol. 51, FP24196. doi: 10.1071/fp24196 39365897

[B33] Li M. Shi. P. Xu C. Ren J. Qu X. (2013). Cerium oxide caged metal chelator: anti-aggregation and anti-oxidation integrated H2O2-responsive controlled drug release for potential Alzheimer’s disease treatment. Chem. Sci. 4, 2536–2542. doi: 10.10.1039/C3SC50697E

[B34] Lian H. Y. Hu M. Liu C. H. Yamauchi Y. Wu K. C. W. (2012). Highly biocompatible, hollow coordination polymer nanoparticles as cisplatin carriers for efficient intracellular drug delivery. Chem. Commun. (Camb). 48, 5151–5153. doi: 10.1039/C2CC31708G 22514015

[B35] Ludwig-Müller J. (2000). Indole-3-butyric acid in plant growth and development. Plant Growth Regul. 32, 219–230. doi: 10.1023/a:1010746806891

[B36] Mahapatra A. Misra B. Banik D. Gupta V. K. Rout D. S. Sahu C. (2024). “Role of nanoparticles on soil microbial community and functionality,” in Nanotechnology applications and innovations for improved soil health (Hershey, USA: IGI Global), 43–56). doi: 10.4018/979-8-3693-1471-5.ch003

[B37] Mandoli C. Pagliari F. S. Forte G. Di Nardo P. Licoccia S. Traversa E. (2010). Stem cell aligned growth induced by CeO2 nanoparticles in PLGA scaffolds with improved bioactivity for regenerative medicine. Adv. Funct. Mater. 20, 1617–1624. doi: 10.1002/adfm.200902363

[B38] McMillan J. Batrakova E. Gendelman H. E. (2011). Cell delivery of therapeutic nanoparticles. Prog. Mol. Biol. Transl. Sci. 104, 563–601. doi: 10.1016/B978-0-12-416020-0.00014-0 22093229 PMC4016803

[B39] Murashige T. Skoog F. (1962). A revised medium for rapid growth and bioassays with tobacco tissue cultures. Physiol. Plant 15, 473–497. doi: 10.1111/j.1399-3054.1962.tb08052.x

[B40] Nuzzo A. Scherman O. Mazzei A. P. Piccolo A. (2014). pH-controlled release of auxin plant hormones from cucurbit [7] uril macrocycle. Chem. Biol. Technol. Agric. 1, 1–8. doi: 10.1186/2196-5641-1-2

[B41] Oliveira H. C. Stolf-Moreira R. Martinez C. Grillo B. R. R. de Jesus M. B. Fraceto L. F. (2015). Nanoencapsulation enhances the post-emergence herbicidal activity of atrazine against mustard plants. PloS One 10, e0132971. doi: 10.1371/journal.pone.0132971 26186597 PMC4506088

[B42] Palard M. Balencie J. Maguer A. Hochepied J. F. (2010). Effect of hydrothermal ripening on the photoluminescence properties of pure and doped cerium oxide nanoparticles. Mater. Chem. Phys. 120, 79–88. doi: 10.1016/j.matchemphys.2009.10.025

[B43] Pereira A. Sandoval-Herrera I. E. Zavala-Betancourt S. A. Oliveira H. C. Ledezma-Pérez A. S. Romero J.. (2017). γ-Polyglutamic acid/chitosan nanoparticles for the plant growth regulator gibberellic acid: Characterization and evaluation of biological activity. Carbohydr. polym. 157, 1862–1873. doi: 10.1016/j.colsurfb.2016.11.027 27987906

[B44] Prasad R. Bhattacharyya A. Nguyen Q. D. (2017). Nanotechnology in sustainable agriculture: recent developments, challenges, and perspectives. Front. Microbiol. 8. doi: 10.3389/fmicb.2017.01014 PMC5476687 28676790

[B45] Reed B. M. DeNoma M. Wada S. Postman J. (2013). “Micropropagation of pear (Pyrus sp.),” in Protocols for micropropagation of selected economically-important horticultural plants, Totowa, NJ 3–18. doi: 10.1007/978-1-62703-074-8_1 23179686

[B46] Safavi K. (2014). Effect of titanium dioxide nanoparticles in plant tissue culture media for enhance resistance to bacterial activity. Bull. Environ. Pharmacol. Life Sci. 3, 163–166.

[B47] Santo Pereira A. E. Silva P. M. Oliveira J. L. Oliveira H. C. Fraceto L. F. (2017). Chitosan nanoparticles as carrier systems for the plant growth hormone gibberellic acid. Colloids Surf B Biointerfaces. 150, 141–152. doi: 10.1016/j.carbpol.2016.11.073 27914250

[B48] Sharma P. Janaagal M. Sheoran A. R. Sharma C. (2024). Nanoparticle innovations in plant systems: enhancing photosynthesis and nutrient dynamics. AJAAR 24, 75–88. doi: 10.9734/ajaar/2024/v24i12574

[B49] Shlar I. Poverenov E. (2021). A nanohybrid layered double hydroxide as an effective carrier for delivery and application of the phytohormone indole acetic acid. Colloids Surfaces B: Biointerfaces 207, 112032. doi: 10.1016/j.colsurfb.2021.112032 34412012

[B50] Socrates G. (1994). Infrared characteristic group frequencies (Chichester, NY, Brisbane, Toronto: John Wiley & Sons). 34–51, 62–7, 80–118, 155–60.

[B51] Srivastava M. Das A. K. Khanra P. Uddin M. E. Kim N. H. Lee J. H. (2013). Characterizations of in *situ* grown ceria nanoparticles on reduced graphene oxide as a catalyst for the electrooxidation of hydrazine. J. Mater. Chem. A. 1, 9792–9801. doi: 10.1039/C3TA11311F

[B52] Strader L. C. Culler A. H. Cohen J. D. Bartel B. (2010). Conversion of endogenous indole-3-butyric acid to indole-3-acetic acid drives cell expansion in Arabidopsis seedlings. Plant Physiol. 153, 1577–1586. doi: 10.1104/pp.110.157461 20562230 PMC2923913

[B53] Sukumaran S. Neelakandan M. Shaji N. Prasad P. Yadunath V. (2018). Magnetic nanoparticles: Synthesis and potential biological applications. JSM Nanotechnol Nanomed. 6, 1068.

[B54] Thangavelu R. M. Gunasekaran D. Jesse M. I. SU M. R. Sundarajan D. Krishnan K. (2018). Nanobiotechnology approach using plant rooting hormone synthesized silver nanoparticle as nanobullets for the dynamic applications in horticulture–an *in vitro* and ex vitro study. Arabian J. Chem. 11, 48–61. doi: 10.1016/j.arabjc.2016.09.022

[B55] Upadhyaya S. Devi M. Sarma N. S. (2023). “Carbon and silver nanoparticles for applications in agriculture,” in Microbiomes for the management of agricultural sustainability (Springer Nature Switzerland, Cham), 297–316. doi: 10.1007/978-3-031-32967-8_18

[B56] Viswanathan D. Christudoss A. C. Seenivasan R. Mukherjee A. (2024). Decoding plant hormesis: cerium oxide nanoparticles and the role of soil EPS in the growth dynamics of allium sativum L. ACS Agric. Sci. Technol. 5, 61–74. doi: 10.1021/acsagscitech.4c00562

[B57] Vitali F. Raio A. Sebastiani F. Cherubini P. Cavalieri D. Cocozza C. (2019). Environmental pollution effects on plant microbiota: The case study of poplar bacterial-fungal response to silver nanoparticles. AMBB 103, 8215–8227. doi: 10.1007/s00253-019-10071-2 31402424

[B58] Wang P. Lombi E. Zhao F. J. Kopittke P. M. (2016). Nanotechnology: a new opportunity in plant sciences. Trends. Plant Sci. 21, 699–712. doi: 10.1016/j.tplants.2016.04.005 27130471

[B59] Woodward AW Bartel B (2005). "Auxin: regulation, action, and interaction." Annals of Botany 95(5), 707–735. doi: 10.1093/aob/mci083 15749753 PMC4246732

[B60] Xu C. Lin Y. Wang J. Wu L. Wei W. Ren J.. (2013). Nanoceria-triggered synergetic drug release based on CeO2-capped mesoporous silica host–guest interactions and switchable enzymatic activity and cellular effects of CeO2. Adv. healthc. Mater. 2, 1591–1599. doi: 10.1002/adhm.201200464 23630084

[B61] Yang Y. Oztekin A. Neti S. Mohapatra S. (2012). Particle agglomeration and properties of nanofluids. J. Nanopart. Res. 14, 1–10. doi: 10.1007/s11051-012-0852-2 22448125

[B62] Zakaria M. B. Belik A. A. Liu C. H. Hsieh H. Y. Liao Y. T. Malgras V.. (2015). PRussian blue derived nanoporous iron oxides as anticancer drug carriers for magnetic-guided chemotherapy. Chem. Asian J. 10, 1457–1462. doi: 10.1002/asia.201500232 25944287

